# #ProtectOurElders: Analysis of Tweets About Older Asian Americans and Anti-Asian Sentiments During the COVID-19 Pandemic

**DOI:** 10.2196/45864

**Published:** 2024-03-29

**Authors:** Reuben Ng, Nicole Indran

**Affiliations:** 1 Lee Kuan Yew School of Public Policy National University of Singapore Singapore Singapore; 2 Lloyd's Register Foundation Institute for the Public Understanding of Risk National University of Singapore Singapore Singapore

**Keywords:** AAPI, anti-Asian hate, anti-Asian, Asian Americans and Pacific Islanders, Asian-American, content analysis, coronavirus, COVID-19, discourse, discriminate, discrimination, discriminatory, Pacific Islander, racial, racism, racist, SARS-CoV-2, social media, tweet, Twitter

## Abstract

**Background:**

A silver lining to the COVID-19 pandemic is that it cast a spotlight on a long-underserved group. The barrage of attacks against older Asian Americans during the crisis galvanized society into assisting them in various ways. On Twitter, now known as X, support for them coalesced around the hashtag #ProtectOurElders. To date, discourse surrounding older Asian Americans has escaped the attention of gerontologists—a gap we seek to fill. Our study serves as a reflection of the level of support that has been extended to older Asian Americans, even as it provides timely insights that will ultimately advance equity for them.

**Objective:**

This study explores the kinds of discourse surrounding older Asian Americans during the COVID-19 crisis, specifically in relation to the surge in anti-Asian sentiments. The following questions guide this study: What types of discourse have emerged in relation to older adults in the Asian American community and the need to support them? How do age and race interact to shape these discourses? What are the implications of these discourses for older Asian Americans?

**Methods:**

We retrieved tweets (N=6099) through 2 search queries. For the first query, we collated tweets with the hashtag #ProtectOurElders. For the second query, we collected tweets with an age-based term, for example, “elderly” or “old(er) adults(s)” and either the hashtag #StopAAPIHate or #StopAsianHate. Tweets were posted from January 1, 2020, to August 1, 2023. After applying the exclusion criteria, the final data set contained 994 tweets. Inductive and deductive approaches informed our qualitative content analysis.

**Results:**

A total of 4 themes emerged, with 50.1% (498/994) of posts framing older Asian Americans as “vulnerable and in need of protection” (theme 1). Tweets in this theme either singled them out as a group in need of protection because of their vulnerable status or discussed initiatives aimed at safeguarding their well-being. Posts in theme 2 (309/994, 31%) positioned them as “heroic and resilient.” Relevant tweets celebrated older Asian Americans for displaying tremendous strength in the face of attack or described them as individuals not to be trifled with. Tweets in theme 3 (102/994, 10.2%) depicted them as “immigrants who have made selfless contributions and sacrifices.” Posts in this section referenced the immense sacrifices made by older Asian Americans as they migrated to the United States, as well as the systemic barriers they had to overcome. Posts in theme 4 (85/994, 8.5%) venerated older Asian Americans as “worthy of honor.”

**Conclusions:**

The COVID-19 crisis had the unintended effect of garnering greater support for older Asian Americans. It is consequential that support be extended to this group not so much by virtue of their perceived vulnerability but more so in view of their boundless contributions and sacrifices.

## Introduction

Not unlike other public health crises, the COVID-19 pandemic brought with it a disconcerting onslaught of racism and xenophobia [1]. The number of anti-Asian hate crimes in the United States quadrupled in 2021, escalating from the already significant uptick it experienced in 2020, when the COVID-19 outbreak was declared a global pandemic [2]. In the Asian American and Pacific Islanders (AAPI) community, those aged 60 years or older accounted for 7.3% of the 2808 self-reported incidents in 2020 [3]. Though not a particularly large figure, underreporting in this community is fairly common [4]. Moreover, older adults have reported being physically assaulted and having to deal with civil rights violations more than the general AAPI community [3]. When the crisis first emerged, older Asian Americans were beleaguered by increased economic insecurity [5] and poorer health outcomes [6] due to a confluence of structural inequities [5].

A silver lining to the COVID-19 pandemic is that it cast a spotlight on a long-underserved group. The barrage of attacks against older Asian Americans galvanized both individuals and organizations into assisting them in various ways, such as by distributing safety whistles and meal vouchers [7]. On Twitter, now known as X, support for them coalesced around the hashtag #ProtectOurElders [4]. The objective of this study is to explore the kinds of discourse surrounding older Asian Americans during the COVID-19 crisis, specifically in relation to the surge in anti-Asian sentiments.

Dating back to the nineteenth century, one of the most pervasive stereotypes of Asian Americans is that they are a high-achieving demographic [8]. While seemingly innocuous, this myth of them as a “model minority” has been criticized as highly problematic. Not only does it run counter to their lived realities—plenty of evidence has exposed the widespread inequalities confronted by various subgroups within the community [8,9]—it also delegitimizes their struggles and feeds the misconception that they require no assistance whatsoever [5].

Racial discrimination is well known to be a key social determinant of health [6,10]. Among Asian Americans in the United States, experiences of discrimination are linked to poorer mental health outcomes, including anxiety, depression, hypertension, and elevated blood pressure [10]. Racism may exacerbate health issues brought about by the aging process, such as the onset of chronic diseases or functional impairment [11], rendering older Asian Americans more susceptible to detrimental health outcomes.

Studies have indicated that social support has a positive impact on both the mental and physical health of older adults [12]. Social support likewise serves as a protective buffer against the negative effects of racial discrimination on one’s health [13,14]. The role of social support may be especially critical for Asian Americans. Although the Asian American populace includes a diverse array of ethnicities, cultures, and languages, collectivism appears to be a cultural orientation shared among many Asian American groups [15]. Evidence revealed that social support improved health outcomes among Asian Americans during the start of the pandemic, when anti-Asian sentiments were rampant [14].

It is widely acknowledged that in Asian societies, attitudes toward older adults are typically informed by values of respect and filial piety [11,16]. Old age bespeaks knowledge and wisdom, and younger people are expected to honor and respect their older counterparts [11]. Despite concerns that such values have eroded, there is evidence that they continue to resonate with Asian Americans [17]. One study concluded that Asian Americans are twice as likely as the general population to care for their parents [18]. Even so, ageism has been discovered to be pan-cultural [19]. A meta-analysis comparing Western and Eastern attitudes toward older adults revealed that Easterners actually harbored more negative views of older adults than Westerners [20]. In this analysis, Western countries included anglophone countries in the West such as Australia, Canada, the United Kingdom, and the United States, as well as Western European countries like Switzerland and France. Eastern countries covered countries in different regions of Asia, such as East Asia, South Asia, and Southeast Asia [20].

First proposed in 2002, the stereotype content model maintains that people stereotype others on the basis of warmth and competence [21]. The dimension of warmth includes qualities such as friendliness and sincerity, while the dimension of competence includes traits such as intelligence and skillfulness [21]. According to the stereotype content model, perceptions of social groups can be categorized into four clusters: (1) warm and competent, (2) incompetent and cold, (3) competent and cold, and (4) warm and incompetent. These 4 combinations of stereotypes produce distinct emotional responses among those who hold them. Groups stereotyped as warm and competent elicit admiration. Those evaluated as incompetent and cold elicit contempt. Groups stereotyped as competent and cold evoke envy. Those evaluated as warm but incompetent evoke pity [21].

A large body of work has evinced that older adults are generally stereotyped as warm but incompetent [21]. Although they elicit feelings of admiration occasionally, they predominantly evoke pity. Evidence attests to the universality of these stereotypes in both individualistic and collectivistic societies [19]. The evaluation of older adults as warm but lacking in competence may lend itself to benevolent ageism—a paternalistic form of prejudice founded on the assumption that older adults are helpless or pitiful [22]. Benevolent ageism has intensified over the course of the pandemic owing to recurring depictions of older adults as an at-risk group [23].

Asian Americans—older or otherwise—are one of the most underresearched ethnic groups in peer-reviewed literature [24,25]. In spite of the discomfiting rise in violence directed at them during the COVID-19 outbreak, discourse surrounding older adults from the Asian American community has escaped the attention of gerontologists. Most social media analyses conducted before and during the pandemic have focused on the discursive construction of the older population as a whole [26-28]. Other social media analyses have concentrated on the general Asian American population [29-31]. This study is therefore conceptually significant in that it is the first to dissect the content of tweets posted about older Asian Americans during the COVID-19 crisis.

At the heart of the concept of intersectionality is the notion that various social positions—such as race, age, gender, and socioeconomic status—interact to shape the types of biases one confronts [32]. From an intersectional standpoint, age and race may converge in ways that worsen the experience of discrimination for older Asian Americans [33]. In addition to being part of a racial group that faces more systemic challenges compared to White people, older Asian Americans also face age-related hurdles [34]. In terms of practical significance, this study serves as a reflection of the level of support being extended to older Asian Americans, even as it provides timely insights that will ultimately advance equity for them.

This study pivots around the following questions: What types of discourse have emerged in relation to older Asian Americans and the need to support them? How do age and race interact to shape these discourses? What are the implications of these discourses for older Asian Americans?

## Methods

### Data Set

We retrieved the data using version 2 of Twitter’s application programming interface (API) [35], which was accessed through Twitter’s academic research product track [36]. Compared to what was achievable with the standard version 1.1 API, the version 2 API grants users a higher monthly tweet cap and access to more precise filters [37].

To build an extensive data set, we collected the tweets using 2 search queries. For both queries, “retweets” were excluded, and only English tweets posted from January 1, 2020, to August 1, 2023, were collated. We excluded retweets to avoid including duplicate content in the data set, which could skew the significance of particular topics. Tweets collected through the first query (n=1549) contained the hashtag #ProtectOurElders. For the second query (n=4550), we gathered tweets that met the following inclusion criteria: (1) contained either the hashtag #StopAAPIHate or #StopAsianHate; (2) included “elder,” “elderly,” “old(er) adult(s),” “old(er) people,” “old(er) person(s),” “senior(s),” “aged,” “old folk(s),” “grandparent(s),” “grandfather(s),” “grandmother(s),” “grandpa,” or “grandma.” The 2 queries yielded a total of 6099 tweets.

**Figure 1:**
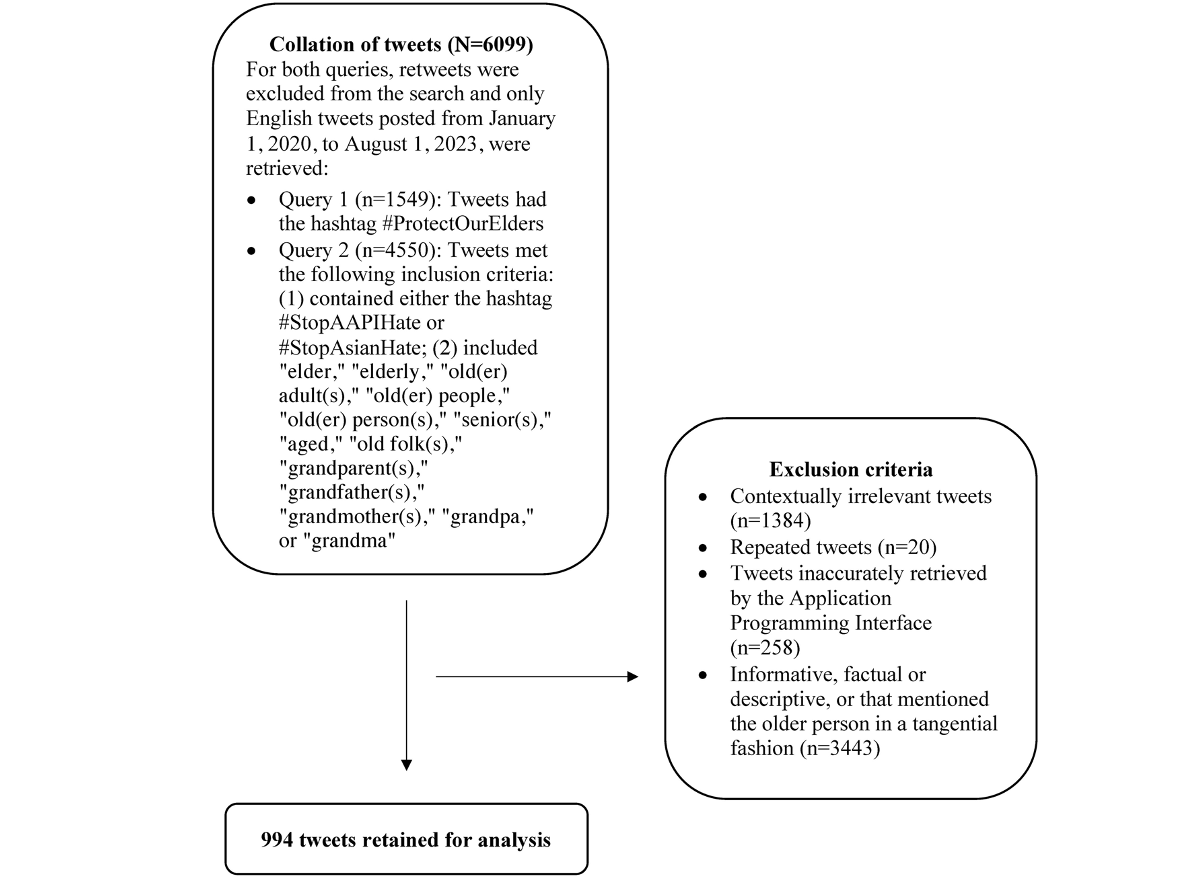
Process of collecting tweets about older Asian Americans in relation to anti-Asian sentiments during the COVID-19 pandemic.

We removed posts that were (1) contextually irrelevant, that is, discussed content not pertaining to anti-Asian attacks, such as tweets related to getting vaccinated to protect older people, or tweets related to protecting older adults from cybercrime (n=1384); (2) repeated in the 2 queries (n=20); (3) incorrectly retrieved by the API, that is, they did not fulfill the inclusion criteria of either search query (n=258); and (4) informative, factual, or descriptive (eg, tweets that were newspaper headlines) or that brought up the older person in a tangential fashion (eg, tweets that mentioned older Asian Americans alongside several other groups; n=3443). After applying the aforementioned exclusion criteria, the data set consisted of 994 tweets. Figure 1 provides a flowchart of the data collection process.

### Tweet Content Coding

Consistent with past research [27,38-41], the codebook was designed through both deductive and inductive modes of reasoning [42]. Analyses led by a directed or deductive approach begin with the identification of an initial set of codes based on previous literature [43]. Conversely, in inductive content analyses, codes are derived directly from the data [43]. We used both deductive and inductive approaches to make sure certain pertinent assumptions guided the analysis while also being aware that new categories would surface inductively [42].

To create a preliminary codebook, we first identified a set of categories based on previous literature regarding the perceptions of older adults in Asia [44]. The content analysis was subsequently conducted in several stages, with each tweet read twice by 2 researchers trained in gerontology to ensure familiarity with and immersion in the data [43]. The goal of the first reading was to ascertain the validity of the initial set of categories as well as to generate codes systematically across the whole data set. Each researcher modified the codebook independently until all variables were refined and clearly defined. During this first reading, a new category was added whenever a post featured a particular trait that could not be suitably coded into any of the existing categories and which was recurrent in the data. During the second reading, the 2 coders had frequent discussions where any discrepancies were reviewed and adjudicated to ensure rigor in the analysis. At this point, both coders discussed what the codes meant, confirmed the relevance of the codes to the research question, and identified areas of significant overlap to finalize the coding rubric.

The percentage agreement between the 2 raters was 92.5% with a weighted Cohen κ of 0.89 (P<.001), indicating high interrater reliability. A total of 4 themes emerged from the whole process. The frequency of each theme was identified after the analysis. As mentioned in past scholarship, categories in a content analysis need not be mutually exclusive, although they should be internally homogeneous (ie, coherent within themes) and externally heterogeneous (ie, distinct from each other) as far as possible [27,45].

### Ethical Considerations

Ethical approval was not deemed necessary for this study, as all the data used were publicly available and anonymized.

## Results

### Summary of Insights From Content Analysis of Tweets

A total of 4 themes emerged from our content analysis of 994 tweets. Half of the posts (498/994, 50.1%) were filed under the theme “vulnerable and in need of protection” (theme 1). Tweets in this theme either singled out older Asian Americans as a group in need of protection because of their vulnerable status or discussed initiatives aimed at safeguarding their well-being. The theme “heroic and resilient” (theme 2) was present in 31.1% (309/994) of the posts. Relevant tweets celebrated older Asian Americans for displaying tremendous strength in the face of attack or described them as individuals not to be trifled with. The theme “immigrants who have made selfless contributions and sacrifices” (theme 3) appeared in 10.2% (102/994) of the posts. Posts in this section referenced the immense sacrifices made by older Asian Americans as they migrated to the United States, as well as the systemic barriers they had to overcome. Theme 4 “worthy of honor” (85/994, 8.5%) consisted of tweets that venerated older Asian Americans. Textbox 1 provides a summary of the themes.

**Description of themes present in tweets about older Asian Americans in relation to anti-Asian sentiments during the COVID-19 pandemic.**

**Vulnerable and in need of protection (498/994, 50.1%)**

- “Isn't it so cowardly that they attack the elderly mostly? Not that violence is acceptable for any age, but to hurt the defenseless only means they got loose screws. #StopAsianHate”
- “Conducting walking patrols everyday to protect our elders and community #StopAAPIHate #HateisaVirus #StopAsianHate #SFChinatown #SafeNeighborhood #ProtectOurElders #TogetherWeCan”

**Heroic and resilient (309/994, 31.1%)**

- “Underestimating the terror wrought by old Chinese ladies with sticks was his first mistake #grannygoals #StopAsianHate”
- “Don't mess with Asian grandmas. But also sad this is happening. #StopAsianHate #StopAAPIHate”

**Immigrants who have made selfless contributions and sacrifices (102/994, 10.2%)**

- “Come to America they said..

It's the land of Opportunities they said...

Feeling so sad seeing this video 2 underage over privileged girls get to do this to a man ,a father ,a grandfather and not even have their identities revealed ...devastating

#MuhammadAnwar #StopAsianHate”

- “These are my grandparents. They came to America to build a new life. (That's my dad on the right wearing a tie.) My grandfather was a very well respected doctor in the Chinese community. America is built on the backbone of hard-working immigrants. #StopAsianHate”

**Worthy of honor (85/994, 8.5%)**

- “What's been shocking to me about these increased attacks on #AAPI is how often the elderly have been the focus. It’s such a shock because one thing that has been common amongst #AAPI culture is the reverence/respect of elders. #StopAAPIHate #StopAsianHate”
- “It really makes me weak and cry seeing videos of those elderly being hit and hurt. We, Asians, value and esteem our elderly. We even live with them in the same house, take care of them. I can't imagine how someone can simply push them. Just like that. #StopAsianHate”

### Theme 1: Vulnerable and in Need of Protection

The vulnerability of older adults was a throughline in this category (498/994, 50.1%). Although concern was directed at the entire Asian American population, older adults were singled out as deserving of more sympathy because of their advanced age. Adjectives commonly used to frame them include “infirm,” “weak,” “defenseless,” and “powerless.” A person described them as lacking “the strength to even unclasp a grip.” Sympathy for older adults was magnified in view of other challenges they had been confronting since the outbreak of COVID-19. For instance, one poster expressed sorrow over how older Asian Americans had to grapple with the “fear of getting attacked” on top of “already [being] really afraid of COVID-19 because it disproportionately affects” them.

What made the act “especially egregious” in the eyes of many was the fact that assailants targeted older adults of all people. Users lambasted attackers for their “coward[ice],” asserting that they should have “picked on someone [their] own size” instead of attacking “people who can’t even defend themselves.” Several posters insisted that it was incumbent upon society to “be watchdogs” for older adults since they are more vulnerable.

A large number of tweets featured a call-to-action aimed at mobilizing members of the Twitter community to assist older Asian Americans. Fundraising campaigns were conducted to raise money for “alarms and pepper spray” for older Asian Americans. Others lobbied for donations to causes that deliver food to this group. The following tweet is one such example: “Wondering how you can support elderly Asians and show you will not tolerate #Asianhate? Join me in making a contribution to @heartofdinner, which brings food to elderly Asians in NYC so they can eat safely in their homes #StopAsianHateCrimes #StopAAPIHate.” The Twitter audience was also invited to escort older persons who walk alone: “United Peace Collaborative protects the #SF Chinatown community with daily walking patrols, providing protection & assistance to the elderly & residents. Please join us & volunteer!”

There were many tweets concerning the suite of initiatives aimed at supporting older Asian Americans. The Yellow Whistle—a campaign involving the distribution of free whistles for Asian Americans to signal danger in the event of an assault—was held up as one such example to “keep older Asian Americans safe.” Select community partners also received plaudits for their “wonderful work in distributing and training use of the alarms to” older persons.

### Theme 2: Heroic and Resilient

Tweets in this theme (309/994, 31.1%) mainly revolved around a high-profile incident in San Francisco in which Xiao Zhen Xie, an older woman of Asian descent, put her assailant on a stretcher in an unexpected turn of events. She earned kudos from the Twitter community for “hold[ing] her ground,” “fighting back,” and sending him “to the hospital with his face bloodied.” Many saluted her for being “feisty,” “resilient,” and “[as] tough as nails,” dubbing her a “hero” who made them feel “#HonoredToBeAsian.” One user used the hashtag “#GrannyGoals,” quipping that the attacker made a “mistake” “underestimating the terror” that “old Chinese ladies” could wreak. Xiao Zhen Xie was also applauded for “refusing to be a statistic” as well as for defying the image of older adults as a group most expect “not to fight back.”

This episode involving Xiao Zhen Xie set in motion a series of tweets in which users warned others not to get on their grandparents’ bad side. A user cautioned that the incident was a lesson to everyone not to “mess with ahjummas, lolas, and all the elderly Asian women.” Another claimed that Asian grandmothers possess a special kind of “Asian grandma strength.” Some took the opportunity to underline the importance of not belittling older adults, with one in particular commenting on how his or her grandparents embodied grit and “toughness” because they “lived through war.”

Besides Xiao Zhen Xie, a few other older Asian Americans were celebrated for their resilience. A Filipina immigrant, Vilma Kari, was lauded for saying she “forgives” and “prays” for her attacker. A handful of tweets focused on a group of older Asian Americans who made the headlines for having filmed a music video in which they condemned the racially motivated acts of violence targeting their community.

### Theme 3: Immigrants Who Have Made Selfless Contributions and Sacrifices

Members of the Twitter community frequently shared stories of their grandparents’ immigration (102/994, 10.2%). A common thread running through these posts was that their forefathers made immense sacrifices, uprooting themselves to move to the United States in order that their children might receive “the best education they can get” and “enjoy a “better future.” A user portrayed his or her grandmother as a “fighter” who “worked two to three jobs” while struggling to acculturate in a new society at a time when she knew “very little English.”

Attention was drawn to how the string of attacks against Asian Americans was ironic given the national ethos of the country commonly touted as the “American dream.” A few posters implied that labeling the United States as a “land of opportunity” was a misnomer: “Come to America,’ they said... ‘It’s the land of opportunities,’ they said...” A user said that the Asian “elderly did not escape communism” only to become a target of racism.

Tweets in this theme also discussed the burden of racism that older Asian Americans had endured before the COVID-19 pandemic. Users commented on their grandparents’ day-to-day experiences of racial discrimination. A handful were dismayed by how their grandparents were survivors of “prejudice and xenophobia” during World War II when they were forcibly relocated to Japanese internment camps. Others bemoaned that their older family members were “imprisoned for being the wrong-colored Americans.” One user deplored the fact that his or her grandfather “could not come to [the United States] because of his race” due to the Chinese Exclusion Act of 1882, a law that suspended Chinese immigration for 10 years and declared Chinese immigrants ineligible for naturalization. Another poster pinpointed how his or her grandfather felt compelled to dress in an “extremely patriotic” manner in order to camouflage his Asian identity and better assimilate into America.

Users considered older Asian Americans as foundational to the growth of America and foregrounded the need to acknowledge that “America is built on the backbone of hardworking immigrants,” who “made 90%” of what society has. Examples of contributions made by those of Asian ancestry include how they “oversaw” the construction of the transcontinental railroad in the “Old West” as well as their “service in the #442RCT (442nd Infantry Regiment)”—a highly decorated infantry regiment that mainly comprised second-generation American soldiers of Japanese descent who served in World War II. One user mentioned Chien-Shiung Wu, a groundbreaking Chinese American physicist whose scientific accomplishments were a core part of “U.S. WW II efforts” and that “helped win Nobel Prizes for Americans,” without which the “country would be so much worse off.” Artworks inspired by “hustling, elderly Asian folks” were also broadcasted under a hashtag that deified them as “#ChinatownGods.”

Several attempts were made to deconstruct the myth of the model minority. Individuals were aggrieved at how the looming specter of anti-Asian violence compounded the plight of older Asian Americans, who had already been dealt multiple blows during the COVID-19 crisis. These posters raised awareness of how many of them are in “precarious living situations” or “working in low-wage jobs.” Some pleaded for the Asian American community to be seen and understood, as captured in the following tweet: “See what’s happening to our elderly and community. Understand us. Understand why no matter how model of a minority we seem to be... we are just like you. #StopAsianHate #StopAAPIHate #StandWithAsians.”

### Theme 4: Worthy of Honor

Many users (85/994, 8.5%) were outraged at how older adults appeared to be prime targets of violence against the Asian American community, perceiving these acts as a flagrant transgression of Asian cultural mores that “revere” them as “the most important people” in society. Some tweets exalted them as wellsprings of “wisdom” and “thoughtful guidance”—one user even likened them to “gold”—to “value and esteem.” Tweets in this theme also alluded to how deference to the older community was practically nonnegotiable in the Asian household. A poster tweeted, “No one should be assaulted, especially the elderly. I grew up respecting my elders. You never even argued with them ... They pass on wisdom.”

Values of collectivism were prized by certain users. These posters made reference to the notion of intergenerational reciprocity by stressing that younger people had an obligation to “protect” the older generation in return. The idea of solidarity was also raised. For instance, some viewed the attack of an older adult—related or otherwise—as an affront to the entire Asian community: “Many are saying ‘she could've been MY grandma.’ To that I say, she is ALL OUR GRANDMAS. Fight hate, love justice, stand with our elders always. #ForTheLoveOfLolas #StopAsianHate #StopAAPIHate #StopAsianHateCrimes.”

## Discussion

### Overview

This study serves as a substantive first step in understanding discourses surrounding older Asian Americans. In our content analysis of tweets posted about the rash of attacks targeting them during the COVID-19 crisis, 4 main discourses surfaced. The first positioned them as “vulnerable and in need of protection” (theme 1). The second characterized them as “heroic and resilient” (theme 2). The third portrayed them as “immigrants who have made selfless contributions and sacrifices” (theme 3), and the fourth extolled them as “worthy of honor” (theme 4).

Our findings demonstrate an outpouring of support for the older Asian American community, which manifested itself in various local initiatives such as the distribution of safety whistles and the delivery of food. Scholars have drawn attention to how social support is particularly crucial for those in their later years [12] as well as those who experience racial discrimination [13,14]. The fact that older Asian Americans are finally being given support and assistance is therefore a step in the right direction.

However, even well-intentioned acts may be met with negative repercussions. In the wake of the COVID-19 crisis, older adults were reduced to a uniform group of at-risk individuals [46]. Assumptions of their vulnerability led to paternalistic behaviors, which denied them their autonomy [23]. Our results indicate that the rise in violence toward older Asian Americans sparked much-needed dialogue regarding their everyday struggles. Nevertheless, an unfortunate corollary is that this may have predisposed them to being recipients of benevolent prejudice on the basis of both age and race. Older Asian Americans may have been viewed as especially defenseless or vulnerable, perhaps more so than the general older population. This was made amply clear in the findings, where half of the tweets branded older Asian Americans as “weak” and “powerless.”

Notwithstanding concerns that Asian values of respect and filial piety have become irrelevant in the face of modernization [17], findings from themes 2-4 show emphatically that older adults retain their revered status, at least among some in the Asian American community. Tweets in theme 2 featured users enthusing over the way Xiao Zhen Xie held her ground when she was attacked in San Francisco, which led to deliberations on the strength and tenacity of older Asian women in general. Discourses of gratitude emerged in theme 3 as users ruminated over the sacrifices their forefathers had made in migrating to the United States, as well as the attendant systemic challenges they had to navigate. Posts in theme 4 indicate that users perceived the violence against older Asian Americans as a contravention of cultural norms, which emphasize the importance of honoring older adults. These provide a countervailing force to the various ageist tropes that came to the fore during the COVID-19 pandemic, such as the #BoomerRemover hashtag, which saw the lives of older people being discounted [27,28].

### Theoretical Contribution and Implications

Findings from this study show that during the COVID-19 pandemic, age and race interfaced in complex ways to shape discourses on older Asian Americans. Specifically, our content analysis demonstrates that the stereotypes of warmth and incompetence, which are often thought to shape evaluations of older adults, cannot be applied indiscriminately to older Asian Americans as a subcategory of the older demographic. Theme 1, which positions older Asian Americans as vulnerable and in need of protection, does indeed align with traditional evaluations of older adults as warm and incompetent. However, the remaining themes celebrate older Asian Americans for their numerous contributions to society, the sacrifices they have made, and their unwavering resilience during the pandemic, all of which challenge the stereotype of incompetence under the stereotype content model. These findings add complexity to the commonly held notion of older adults as a pitiful social group by highlighting that older Asian Americans evoke not just pity but also admiration. The stereotype content model should therefore be expanded or modified in a way that accounts for attitudes toward older adults of different ethnicities.

Additionally, gerontological scholarship would benefit from a cross-cultural analysis of benevolent ageism. At present, little is known about how displays of benevolent ageism are affected by cultural norms of parental respect and filial piety and the extent to which these norms affect one’s perception of an older adult’s competence. Several studies have been conducted to make sense of ageism in different cultures [47,48], but there has been limited research on the cross-cultural differences in benevolent ageism specifically. The ways in which evaluations of older Asian Americans may be complicated by the deeply ingrained myth of the model minority as well as the pandemic-induced rise in anti-Asian hate are important avenues for future study.

This study has a number of implications for policy and practice. First, although care toward one’s parents or grandparents is not the prerogative of Asians [49], Asia’s adherence to collectivism nonetheless offers a useful learning point for the West. Many of the posters were Asian Americans, who held older adults in high regard, whether related or otherwise. Fostering a cultural emphasis on solidarity and interconnectedness in the West may promote respect not only for one’s parents but also for older adults outside of one’s family [44]. Second, ongoing efforts to reframe aging [50] could highlight the need to respect older adults, not in a way that advances their supremacy or absolves them from wrongdoing, but in a way that teaches society to view them as people whose experience may render them wise and worth learning from. Educators could also incorporate lessons on age-related stereotypes in schools to guard against the formation of ageist beliefs [51].

Third, current moves to redress the longstanding omission of Asian American history from national curricula [52] should ensure that students in every state are taught about the sacrifices, struggles, and contributions of older Asian Americans. Public campaigns could be organized as well to raise awareness of the aforementioned. This will help counter the myth of the model minority and get more people to acknowledge older Asian Americans as a significant part of America’s social fabric. Fourth, our findings underscore the need to reflect on the diversity of the older population in terms of socioeconomic status. Older adults—particularly those from the baby boomer generation—have been stereotyped as having made significant financial gains compared to their predecessors, at times even seen as stealing resources from the young [53]. However, as highlighted by some of the Twitter users as well as scholars, many older Asian Americans are in dire economic straits [5]. Rectifying the structural inequities that have contributed to their immiseration should hence be a key component of the agenda moving forward.

There are limitations inherent in this study. First, we acknowledge that Twitter users might not be representative of the wider population and that only publicly available tweets were included in the data set. Some of the users whose tweets were included in the study appeared to be Asian Americans, who are likely to be more passionate about supporting individuals in their community. Relatedly, as we did not collect information regarding users’ demographics—not all users publish demographic information, and there are certain limitations to using publicly provided demographic information on social media [54]—we could not contextualize the motivations of those whose tweets were included in the analysis. Ultimately, social support for older Asian Americans—whether from the Asian American community or society as a whole—has important implications for their well-being [14]. Subsequent research could focus on conducting interviews among individuals from different ethnic groups to tease out any differences in the level of support extended to older Asian Americans.

Second, we queried the hashtag #StopAAPIHate as a way to understand sentiments toward Asian Americans, even though the term “AAPI” refers to 2 different racial groups: Asian Americans and Pacific Islanders. As the tweets analyzed paid more attention to older Asian Americans, we were not able to offer insight into the types of discourses that emerged in relation to older Pacific Islanders. Future studies are needed to expound on such discourses. Third, it is vital to highlight that both the Asian American community and the older population are heterogeneous. The Asian American community encompasses numerous ethnicities, all with distinct languages, cultures, immigration histories, values, and beliefs [34]. The older demographic, too, is a diverse group composed of people with vastly different health trajectories [55]. Given the brevity of the tweets uploaded, we were unable to assess how discourses on older Asian Americans vary across different ethnicities. Finally, we collected only textual data, although tweets often contain visual elements such as photos, videos, and GIFs. This is a drawback that can be overcome in the future when multimodal techniques are developed to analyze both textual and visual content on Twitter.

Another direction for future inquiry involves an assessment of how discourses surrounding older Asian Americans have changed over time. The level of support shown to this group is likely to fluctuate over time, depending on the frequency at which anti-Asian attacks are reported in the news as well as other types of news being covered. Sentiment and narrative analyses [56-58] could be performed to glean such insights.

### Conclusion

Even as older Asian Americans contended with a rise in racism alongside other struggles during the COVID-19 pandemic, our findings reveal that the crisis had the unintended effect of garnering greater support for this group. In the future, it is important that support be extended to older Asian Americans not so much by virtue of their perceived vulnerability but more so in view of their boundless contributions and sacrifices.

## Abbreviations

AAPI: Asian American and Pacific Islanders
API: application programming interface

## Data Accessibility

Data are publicly available on Twitter [59].
